# Integrated care pathways on dementia in Italy: a survey testing the compliance with a national guidance

**DOI:** 10.1007/s10072-019-04184-9

**Published:** 2019-12-13

**Authors:** Giuseppe Gervasi, Guido Bellomo, Flavia Mayer, Valerio Zaccaria, Ilaria Bacigalupo, Eleonora Lacorte, Marco Canevelli, Massimo Corbo, Teresa Di Fiandra, Nicola Vanacore

**Affiliations:** 1 National Center for Disease Prevention and Health Promotion, National Institute of Health, Via Giano della Bella no. 34, 00162 Rome, Italy; 2 Department of Biomedicine and Prevention, Hygiene and Preventive Medicine School, University of Rome Tor Vergata, Rome, Italy; 3 Department of Human Neuroscience “Sapienza”, University of Rome, Rome, Italy; 4 Department of Neurorehabilitation Sciences, Casa Cura Policlinico, Via Dezza 48, 20144 Milan, Italy; 5 General Directorate for Health Prevention, Ministry of Health, Rome, Italy

**Keywords:** Dementia, Cognitive disorders, Care pathway, Care coordination, Integration of care

## Abstract

Dementias are chronic, degenerative neurological disorders with a complex management that require the cooperation of different healthcare professionals. The Italian Ministry of Health produced the document “Guidance on Integrated Care pathway for People with Dementia” (GICPD) with the specific objective of providing a standardized framework for the definition, development, and implementation of integrated care pathways (ICP) dedicated to people with dementia. We searched all available Italian territorial ICPs. Two raters assessed the retrieved ICPs with a 2-point scale on a 43-item checklist based on the GICPD. Only 5 out of 21 regions and 5 out of 101 local health authorities had an ICP, with most ICPs having a moderate compliance to the GICPD, in particular for the items referring to the development and implementation of the care pathways. A low to moderate inter-rater agreement was observed, mainly due to a lack of standardized models to describe ICPs for dementias. Results suggest that policy- and decision-makers should pay more attention to the GICPD when producing ICPs. The direct communication with clinicians, and the implementation of more precise and appropriate clinical outcomes, could increase the involvement of clinicians, whose participation is crucial to guarantee that ICPs meet needs of patients and their carers.

## Background

Dementia is a chronic, progressive syndrome affecting both cognitive and functional abilities. Though being most prevalent in older people, dementia cannot be considered part of the normal ageing process [1].

In the last years, the World Health Organization (WHO) urged member States to consider dementia as a public health priority. In particular, the WHO estimates that almost 50 million people are currently living with dementia worldwide, with a cumulative incidence of about 1 new case diagnosed every 3 s, and a prevalence expected to double in the next 10 years [1, 2]. These numbers highlight how relevant is the public health impact of this condition, considering also its huge economic burden, amounting to US$ 800 billion per year, and the absence of specific diseases-modifying treatment [2]. The current strategies for the management of people with dementia (PwD) include a timely diagnosis, the promotion of accurate and adequate initiatives aimed at preventing the onset of dementia, the early detection of physical or psychological conditions associated with the dementia (e.g. episodes of behavioural and psychological symptoms of dementia**)**, and activities to support families and caregivers [3].

An adequate management of dementia requires a multidisciplinary and multi-professional approach, with a continuous collaboration between general practitioners (GPs), specialized medical staff (e.g. neurologists, psychiatrics, geriatricians), psychologists, social operators, and other healthcare professionals (e.g. physiotherapist, speech therapists, cognitive rehabilitators). This cooperative approach aims at improving the quality of the care and, as a consequence, at improving the quality of life of PwD and their relatives and/or carers [4].

An increasing number of studies assessing the availability of specialized healthcare services [5–7] suggests that the implementation of an “integrated care pathways” (ICPs), resulting from a merge of several care models, can be a crucial element in achieving the optimal management of people with complex chronic disorders, such as PwD. Italy has been using this approach since the implementation of the Italian National Healthcare Plan, which requires the adoption of ICPs, care pathways, or clinical pathways for the management of chronic and complex diseases, such as dementia [8]. The European Pathway Association (EPA) defines an ICP as “a complex intervention for the mutual decision making and organization of care processes for a well-defined group of patients during a well-defined period” [9]. The essential elements of an ICP are the inter-professional teamwork, and the communication systems used among the different professional workers who cooperate within the ICP, as required by the complexity of the target chronic condition.

Both the “integration of care” and the “coordination of care” are considered key elements also for the development and implementation of National Dementia Plans (NDP), as reported in the Alzheimer Disease International (ADI) checklist [10]. The NDP is a public health document aimed at defining the strategic framework, the objectives, and the actions required for an appropriate management of people with dementia [11].

The Italian Ministry of Health (IMoH) requires each regional and local administration to implement the indications included in the Italian NDP [12, 13]. One important action included in the Italian NDP is the development and implementation of specific networks including different health professionals. This network should be included within an appropriate, high-quality ICP. Therefore, in 2017, the IMoH produced the “Guidance on Integrated Care Pathway for People with Dementia” (GICPD) to support health authorities in the development of ICPs dedicated to PwDs. The GICPD includes the main requirements for defining appropriate ICPs on dementia, and categorizes them in the following three domains: executive aspects, essential elements, and development of a pathway. Moreover, the table of contents of the GICPD can be used as an outline of what is required within each local ICP documents [4]. Since 2014, when the NDP was published, only a small number of ICPs on dementia have been produced by the Italian health authorities.

The aim of the present study is to test the compliance of the official documents describing the ICPs produced so far by the Italian health authorities, with the GICPD produced by the IMoH.

## Materials and methods

We screened the official documents reporting and describing all available Italian ICPs for dementia produced by regions and Local Health Authorities (LHA). We browsed all the websites of health authorities and downloaded all official documentation related to the development and implementation of ICPs for PwD. We included in this study all ICPs produced by regions and LHA currently adopted in their area of reference up to June 2019. Documents reporting only generic directions on how to produce a local ICP, and documents that did not provide the entire care pathway, were excluded.

Based on the table of contents of the official GICPD, we created a checklist to test the compliance of ICP documents to the GICPD [4]. The checklist included 43 items addressing the following three domains (see Table 1): executive, essential elements, and development and implementation. The executive domain included items referring to the formal aspect of the care pathway, and, in particular, to the official components (i.e. promoter, coordinator, and customer), teamwork, and diffusion of the document. The essential elements domain included items referring to the main components required in a care pathway, such as the presence of multidisciplinary and multi-professional teams, the active involvement of patients and carers, and the continuity of care. The development and implementation domain refers to the operative part of the document, providing the definition of all ICP phases, and the indications for the monitoring system [4].

**Table 1:** Checklist used to test the performance of integrated care pathways for dementia

Domain 1. Executive (scoring 0–15)	
1.1. Demographical analysis of target population	
1.2. Recognize the scientific evidence on integrated care pathways	
1.3. Presence of the analysis of the local legislation	
1.4. Presence of the appointment of the customer service	
1.5. Presence of the appointment of the promoter	
1.6. Presence of the appointment of coordination team	
1.7. Presence of a multidisciplinary teamwork	
1.8. Presence of a multi-professional teamwork	
1.9. Presence of general practitioner as member of teamwork	
1.10. Presence of patient’s associations as member of teamwork	
1.11. Presence of list of document to provide based on the media	
1.12. Transmission of document between healthcare workers	
1.13. Transmission of the document to the general population	
1.14. Presence of the date of creation	
1.15. Presence of the date of revision and update	
Domain 2. Essential elements (scoring 0–14)	
2.1. Active engagement of patients and their relatives	
2.2. Involvement of all the services dedicated to subject with cognitive disorders	
2.3. Involvement of all the healthcare workers specialized in cognitive disorders	
2.4. Presence of the contact person with telephone number	
2.5. Presence of the pivotal role of general practitioner	
2.6. Presence of specialized communication system with patients and their relatives	
2.7. Presence of specialized communication system between the operative healthcare workers	
2.8. Institution of specialized informative system	
2.9. Presence of healthcare worker with the role of connector	
2.10. Presence of the service of “counselling”	
2.11. Adoption of formalized and standardized guidelines or operative protocols	
2.12. Presence of facilitator (professional worker or technical team)	
2.13. Presence of the flow diagram of the integrated care pathway	
2.14. Presence of the table of task of the integrated care pathway	
Domain 3. Development and implementation (scoring 0–14)	
3.1. Definition of the type of pathway	
3.2. Presence of the analysis of the patient’ needs	
3.3. Presence of the analysis of the current management system	
3.4. Description of the gold standard of the pathway	
3.5. Definition of the expected outcome	
3.6. Definition of the updating services and of the changing area	
3.7. Presence of the results of the pilot study	
3.8. Definition, development, and implementation of the local care pathway	
3.9. Presence of the monitoring systems of the integrated care pathway	
3.10. Presence of qualitative indicators	
3.11. Presence of organizational indicators	
3.12. Presence of the process indicators	
3.13. Presence of the outcomes indicators	
3.14. Presence of economic indicators	

Two independent reviewers assessed the compliance of local ICPs to the national guidance applying a two-point scale (i.e. presence or absence) to each item of the checklist, using the following scoring system:

- Domain 1—executive, 15 items with a score ranging from 0 to 15
- Domain 2—essential elements, 14 items with a score ranging from 0 to 14
- Domain 3—development and implementation, 14 items with a score ranging from 0 to 14
- Total score (calculated by summing the individual scores from the 3 domains), ranging from 0 to 43

Descriptive statistical analyses were performed using the Data Analysis Tool of MS Excel, including means ± standard deviations (SD), medians, interquartile ranges (IQR), minimum and maximum scores for each domain, and total score. Statistical differences were analyzed using the *t* test with a significance level set at *p* < 0.05.

The quality of considered ICP documents was assessed based on both their compliance to the national guidance checklist, and on their clarity. The clarity of the documents was defined by measuring the consistency between the assessments performed by the two independent raters. Consistency was analyzed using the intra-class coefficient (ICC), and inconsistencies between raters were resolved by consensus. Values of ICC lower than 0.5 were considered poor inter-rater reliability (IRR), values ranging between 0.5 and 0.75 were considered moderate IRR, values ranging between 0.75 and 0.9 were considered good IRR, and values higher than 0.90 were considered excellent IRR [14]. As different formulas for calculating the ICC are available, we calculated ICCs and their 95% confidence intervals based on a single rating (*k* = 2), absolute-agreement, 2-way random-effects model using SPSS statistical package version 23 (SPSS Inc., Chicago, IL). Moreover, we tested whether the ICC value was different from zero using the F test. A value lower than 0.05 was considered statistically significant.

## Results

Overall, we screened 13 official documents reporting on the local implementation of ICPs for PWD in Italy. Of these, 8 documents were produced by Italian regions (i.e. Piemonte, Trento, Veneto, Liguria, Emilia-Romagna, Marche, Molise, and Campania) and 5 by LHA (i.e. Brescia, Milano, Treviso, Umbria 1, and Roma 3). The application of the predefined inclusion and exclusion criteria led to the exclusion of 2 documents from the regional authorities of Liguria and Campania, respectively [15–28].

**Fig. 1:**
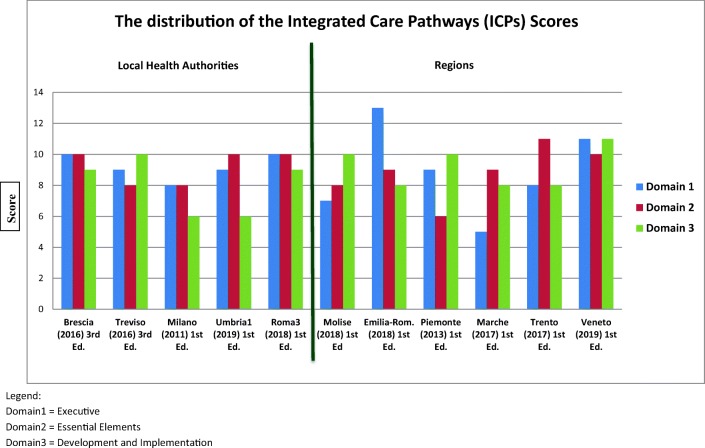
The distribution of the integrated care pathways (ICPs) scores. Domain 1, executive. Domain 2, essential elements. Domain 3, development and implementation

**Fig. 2:**
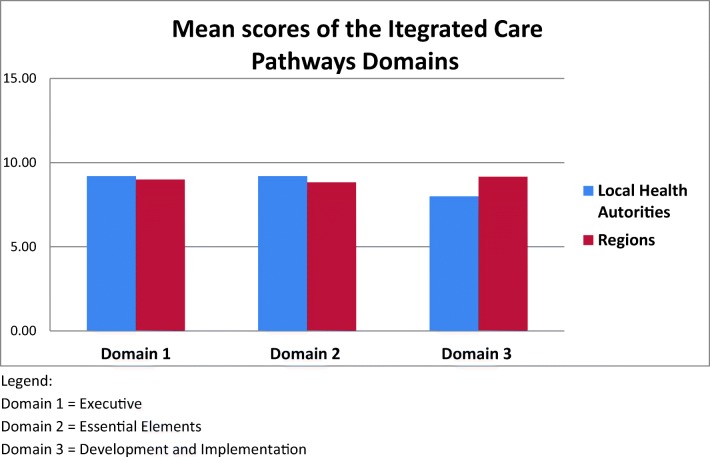
Mean scores of the integrated care pathways domains. Domain 1, executive. Domain 2, essential elements. Domain 3, development and implementation

Overall, the compliance of considered ICPs to the checklist was moderate, with ICPs obtaining a mean score of 25.72 ± 2.8, ranging from 22 (Milano and Marche) to 32 (Veneto), and a median of 26 with an IQR between 24 and 27. The distribution of the scores obtained by each ICP for each domain is reported in Fig. 1. A moderate compliance to the checklist was observed for all the three domains, all of which presented a median score of 9 with an IQR between 8 and 10. In particular, the mean score for domain 1 was 9.18 ± 2.1, the mean score for domain 2 was 9.0 ± 1.2, and the mean score for domain 3 was 8.63 ± 1.6 (Fig. 2).

No statistical differences were observed between the ICPs produced by LHAs and the ICPs produced by regions (*p* = 0.23). The mean total score obtained by ICPs produced by LHA was 25, ranging from 22 (Milano) to 27 (Brescia and Roma3), while the mean total score obtained by regional ICPs was 26.33, ranging from 22 (Marche) to 32 (Veneto). Remarkably, for domain 3, the highest mean score was obtained (9.17 ± 1.32) by regional ICPs, while the lowest mean score (8.0 ± 1.87) was obtained by the ICPs produced by LHAs.

A further, in depth, analysis of the single items of the checklist allowed gathering some additional information. All included ICPs included some specific issues, such as a demographic analysis of the target population (item 1.1), the presence of multi-professional teams (item 1.8), the activation of “counselling” for PwD and their relatives and/or carers (item 2.10), an analysis of requirements and context (items 3.2 and 3.3), and the development and implementation of care pathway (items 3.8). The lowest scores were assigned to the items related to the use of economic indicators (item 3.14) and qualitative indicators (item 3.10), and to the official designation of a promoter of ICP (item 1.5) and/or of coordination team (item 1.6).

Four out of the 11 included ICPs (36.3%) considered the use of informative system, 6 ICPs (54.5%) included outcome indicators in the monitoring system, 9 ICPs (81.8%) provided process indicators, and 7 (63.6%) provided organization indicators (Table 2). In particular, out of the 6 ICPs monitoring the outcome indicators, 4 regional ICPs provided indicators based on the clinical evaluation of subjects with cognitive disorders (Table 3). Three of these (Emilia-Romagna, Veneto, and Trento) considered a clinical outcome based on the current guidelines for the evaluation of the severity of dementia [29–31].

**Table 2:** Focus on the presence of informative system and indicators

Item	Brescia	Treviso	Milano	Umbria1	Roma3	Molise	Emilia-Romagna	Piemonte	Marche	Trento	Veneto	N/tot (%)
2.8	X	X	X	X	X	✓	✓	X	✓	✓	X	4/11 (36.3%)
3.10	X	✓	X	X	X	X	X	X	X	X	X	1/11 (9%)
3.11	X	X	X	✓	✓	✓	✓	✓	X	✓	✓	7/11 (63.6%)
3.12	✓	✓	X	✓	✓	✓	✓	✓	X	✓	✓	9/11 (81.8%)
3.13	X	X	X	X	✓	✓	✓	✓	X	✓	✓	6/11 (54.5%)
3.14	X	X	X	X	X	X	X	X	X	X	X	0 (0%)

**Table 3:** List of the outcome indicators of the 11 selected integrated care pathways

	Clinical outcome indicators	Non-clinical outcome indicators
Local health authorities		
Umbria 1	-	-
Roma 3	-	- Hospitalization rate for ordinary access - Institutionalization rate.
Milano	-	-
Brescia	-	-
Treviso	-	-
Regions		
Emilia-Romagna	- % of MCI patients with neuropsychological evaluation at diagnosis. - % of patients aged < 65 years and neuropsychological evaluation at diagnosis. - % of patients with dementia in drug therapy with neuroleptics. - % new cases with anti-dementia drugs according to the Regulatory Italian Authority for Drugs (AIFA note 85). - % of patients with anti-dementia drugs according to the Regulatory Italian Authority for Drugs (AIFA note 85).	- Hospitalization rate for ordinary access in cases of DRG code 429 (calculated per 100,000 inhabitants). - Hospitalization rate for ordinary access of BPSD cases.
Marche	–	–
Molise	–	- Increased number of timely diagnosis.
Piemonte	- Total number of liquor examination provided/number of cases with suspected cognitive decline.	- Detected cases with timely diagnoses/number of first access.
Veneto	- Number of neuropsychological evaluations carried out for diagnostic purposes/Number of first visits per year stratified by MMSE values.	- Total number of first visits per year for patient with suspected cognitive disorder.
Trento	- Number of cases sent by GP to the CCDD according to GPCog test per year.	- Number of assessments for PwD made by multidimensional evaluation unit per year.

*MCI*, mild cognitive impairment; *BPSD*, behavioural and psychological symptoms of dementia; *AIFA*, Agenzia Italiana del Farmaco; *DRG*, diagnosis related group; *CCDD*, Center for Cognitive Disorders and Dementia; *GP*, general practitioner; *GPCog*, general practitioner cognitive examination test

The analysis of consistency between raters showed a poor to moderate between-rater agreement for total scores, with an ICC of 0.43 (Table 4). In particular, we observed a moderate agreement for domain 3, with an ICC value of 0.58, and a poor agreement on both domain 2 and domain 3 with an ICC value of 0.33.

**Table 4:** Agreement between the two researchers in the assessment of the 11 selected integrated care pathway

Measures	ICC	95% confidence interval		*p* value*
		Lower limit	Upper limit	
Total	0.43	0	0.81	0.008
Domain 1 (executive)	0.33	0	0.73	0.064
Domain 2 (essential elements)	0.33	0	0.74	0.028
Domain 3 (Development and implementation)	0.58	0	0.86	0.029

*ICC*, intra-class correlation coefficient

*F test with true value 0

## Discussion

The “integration of care” approach is a key element for the effective and sustainable clinical management of chronic and disabling diseases [12]. Being the NDP a strategic framework for the management of PwD, the ADI suggests how some objectives of this plan should address specific actions aimed at planning, developing, and implementing specialized ICPs for the management of subjects with cognitive disorders [9].

However, the ADI has recently pointed out some crucial operational issues that are still affecting the implementation of NDPs in various countries [10]. One of these is the allocation of resources and funds, since many countries did not provide specific budgets for the implementation of their NDP. Though Italy is among these countries, the second objective of the Italian NDP requires the development of a network among the existing health services dedicated to dementia.

The implementation of the Italian NDP [11] started in 2014, and two official documents were subsequently released in 2017 (“Guidance on Integrated Care pathway for People with Dementia” and “Guidance of the application of the informative system for the Dementia”) whose content was to be implemented by all Italian health authorities [4, 32, 33]. To date, only 6 out of 21 (29%) regions and 5 out of 101 (5%) Italian LHAs have developed and implemented a specific ICP for the management of PwD.

In our analysis, the low to moderate compliance with GICPD observed across all included ICPs both in the overall scores and in the specific scores for each checklist domain underlines the need for more appropriate ICPs for the management of PwD, and a deep revision of the currently available ones. In particular, the items referring to the more formal aspects of the document and to the institution of a monitoring system were almost completely omitted in the official documents. This could reflect an inadequate level of attention by part of policy-maker to the executive aspects of the documents and to the monitoring of the proposed pathways. The absence of executive implications of the document can lead to an inappropriate or scarce implementation of the pathway, as the pathway might be considered optional and not mandatory. Moreover, the absence of a qualitative evaluation of the pathways can produce an ICP that is not tailored on the actual needs of PwD nor on the expectations of healthcare professionals. Finally, the absence of an economic evaluation can prevent a more rational reallocation of the already few available resources.

Moreover, when focusing on the clinical outcomes, only 3 regional ICPs considered specific and adequate indicators. In fact, the presence of adequate indicators was associated with higher levels of appropriateness of the ICPs in terms of adequate clinical definition of the target population, and adequate management of cases based on disease severity, especially when applying of the best available therapeutic options. The presence of clinical outcome indicators was also associated with an active involvement of clinicians, who are immediately able to assess the relevance of the pathway based on the current evidence-based guidelines. Thus, we strongly recommend improving this aspect in all the current available ICPs and in those that are still under development.

A recent survey carried out among Italian clinicians pointed out that 378 out of 785 physicians (48.2%), from 3 different Italian regions, consider the systematic use of guidelines or care pathways as the best solution to reduce or avoid the recourse to defensive medicine [34]. In fact, the recent, dramatic increase clinicians adopting a defensive medicine approach could have been responsible for the parallel increase in the costs linked to medical malpractice and/or claims from insurance companies. This, along with the progressively lower budget allocated to healthcare, might have a negative impact on the burden and sustainability of national public health services [34, 35]. Therefore, policy-makers and decision-makers must take into consideration the need to improve and support the implementation of good practices, in order to provide the most appropriate and adequate healthcare services. Guaranteeing the most appropriate and adequate services is only possible through the implementation and application of evidence-based guidelines and pathways, that should be produced by specifically created scientific committees or expert panels [36].

Designing and producing high-quality public health documents require some important technical skills, in particular when dealing with the development process of an ICP. The first step for the production of ICPs is collecting and summarizing the highest quality guidelines, a process that requires gathering all evidence-based literature, including systematic reviews and meta-analysis, aimed at answering to specific clinical questions. Results from the assessment and summary of gathered evidence should then be adapted to the specific target clinical services dedicated to dementias. Thus, the implementation and application of care pathways in clinical practice imply the adoption of specific guidelines. This can lead to relevant benefits in terms of efficiency (i.e. reduction of costs, reduced length of hospitalizations, improved quality of life, lower number of hospitalizations, and lower frequency of complications) and appropriateness of care [36, 37].

The low level of agreement between the two raters was considered reflecting a low level of clarity of the ICP documents. Such low clarity could be associated with a lack of standardized and shared models on how to design and write an ICP. Clarity is a crucial aspect in all official public health documents, as it can prevent personal and subjective misinterpretations of the contents, which could lower the quality of the provided health services.

Our searches retrieved a very low number of available official ICPs for dementia. This can be interpreted as reflecting a lack or scarce interest by health authorities towards appropriate strategies for management of these severe and complex conditions. The lack of available territorial ICPs, along with their low compliance to the GICPD, does not meet the requirements stated within the NDP. We also observed a wide difference in the proportion of ICPs produced by regions (29%) and the ICPs produced by LHAs (5%). Moreover, the low mean scores obtained in domain 3 show that the ICPs produced by LHAs are usually more formal than operative documents, though LHAs are considered the operational units of the Italian National Health System. Therefore, health authorities should acknowledge all existing documents, and both promote the production of new high-quality documents and revise the existing ones according to the current guidance, in particular for the ICPs produced by LHAs.

In conclusion, the production of high-quality ICPs on the management of dementias, based on the guidance issued by the Italian MoH, should be promoted and strongly recommended. The activities for increasing the availability of these documents should start with a survey of all operative services dedicated to PwD, followed by the definition of a specific model of network tailored on the needs of both patients and their families and/or caregivers. To achieve an adequate level of appropriateness, all produced ICPs should then be monitored constantly and carefully, using precise, validated indicators, including the ones aimed at assessing the quality of services and controlling costs. Creating a precise monitoring system could allow clinicians to adapt the outcomes of the pathway to the patient’s needs. However, policy-maker, on their part, should implement different clinical outcome indicators, and strengthen the direct communication with clinicians, to prevent the feeling of neglect that is often reported by both clinicians (i.e. specialists and general practitioners) and all health professional involved in the ICPs. Only this, along with a rigorous, systematic approach can allow improving the quality of existing and future ICPs, ensuring that all health professionals provide the most appropriate and adequate health services, thus improving the quality of care and quality of life of PwD and their families and carers.
